# IL-4-induced caveolin-1-containing lipid rafts aggregation contributes to MUC5AC synthesis in bronchial epithelial cells

**DOI:** 10.1186/s12931-017-0657-z

**Published:** 2017-09-20

**Authors:** Yu Xia, Peng-Cheng Cai, Fan Yu, Liang Xiong, Xin-Liang He, Shan-Shan Rao, Feng Chen, Xiang-Ping Yang, Wan-Li Ma, Hong Ye

**Affiliations:** 1 Department of Pathophysiology, School of Basic Medicine, Tongji Medical College, Huazhong University of Science and Technology, Wuhan, Hubei 430030 China; 2 Department of Clinical Laboratory, Union Hospital, Tongji Medical College, Huazhong University of Science and Technology, Wuhan, Hubei China; 3 Department of Respiratory and Critical Care Medicine, Union Hospital, Tongji Medical College, Huazhong University of Science and Technology, Wuhan, Hubei China; 4 Department of Forensic Medicine, Nanjing Medical University, Nanjing, Jiangsu China; 5 Department of Immunology, School of Basic Medicine, Tongji Medical College, Huazhong University of Science and Technology, Wuhan, Hubei China; 6 Key Laboratory of Pulmonary Diseases, Ministry of Health of China, Wuhan, Hubei China

**Keywords:** IL-4, Bronchial epithelial cells, Lipid rafts, Intracellular Ca^2 +^, MUC5AC, Asthma

## Abstract

**Background:**

Mucus overproduction is an important feature of asthma. Interleukin (IL)-4 is required for allergen-induced airway inflammation and mucus production. MUC5AC gene expression is regulated by transcript factors NF-κB. The intracellular Ca^2+^ ([Ca^2+^]_i_) signal is required for activation of NF-κB. The transient receptor potential canonical 1 (TRPC1) channel has been shown to contribute for agonist-stimulated Ca^2+^ influx in some types of cells. However, the relationships among IL-4, TRPC1 and mucus overproduction in bronchial epithelial cells (BECs) in asthma are poorly understood.

**Methods:**

BECs were isolated from large bronchial airway of rats and used as cell model. To present changes of lipid raft, caveolin-1 and TRPC1, immunofluorescence staining and sucrose gradient centrifugation were performed. [Ca^2+^]_i_ was measured after loading with Fura-2. NF-κB activities were measured by an ELISA-based assay. MUC5AC mRNA and protein levels were detected by real-time quantitative RT-PCR, ELISA analysis and immunofluorescence staining respectively.

**Results:**

IL-4 induced Ca^2+^ influx in BECs, and this was blocked by a Ca^2+^ influx inhibitor (2-APB). 2-APB also prevented MUC5AC protein synthesis induced by IL-4. Depletion of extracellular Ca^2+^ resulted in partial decrease in expression of MUC5AC in IL-4 treated cells. NF-κB rather than STAT6 activation mediated IL-4-induced MUC5AC protein synthesis. Then the mechanism of Ca^2+^ influx was investigated. Immunofluorescence staining and sucrose gradient centrifugation revealed that caveolin-1-containing lipid rafts aggregation was involved in TRPC1 activation and Ca^2+^ influx in BECs. Lastly, the data revealed that blocking lipid rafts aggregation exactly prevented Ca^2+^ influx, NF-κB activation and MUC5AC synthesis induced by IL-4.

**Conclusions:**

Our results indicate that IL-4-induced caveolin-1-containing lipid rafts aggregation at least partly contributes to MUC5AC synthesis in BECs.

**Electronic supplementary material:**

The online version of this article (10.1186/s12931-017-0657-z) contains supplementary material, which is available to authorized users.

## Background

Mucus overproduction is an important feature of asthma [1]. Excessive accumulation of airway mucus leads to the formation of mucous plugs that reduce the effective airway diameter and increase airway resistance. Mucins are glycoproteins that provide mucus with its viscoelasticity. MUC2, MUC4, MUC5AC, and MUC5B proteins are considered to be airway mucins. In asthma, there is over-expression of the major mucin glycoprotein, MUC5AC [2, 3].

Allergic pulmonary inflammation and airway hyper-reactivity in most cases of asthma is closely related in T helper type 2 cells (Th2) responses [4]. Th2 cells predominantly secrete cytokines interleukin (IL)-4, IL-5, IL-9 and IL-13, which play a central role in the pathophysiology of asthma. These type 2 cytokines are targets for pharmaceutical intervention of asthma. Studies showed that IL-4 is required for allergen-induced airway inflammation and mucus production [5–7]. MUC5AC expression is increased in mice over-expressing IL-4 compared to transgenic-negative control [7]. IL-4 binds to IL-4 receptor in airway cells and can trigger STAT6 and NF-κB signal pathway which is likely to be involved in the regulation of MUC5AC expression.

*MUC5AC* gene expression is regulated by transcript factors NF-κB and STAT6 [8–10]. NF-κB is involved in cytokine-induced MUC5AC expression. IL-17 promoted NF-κB translocation which subsequently binds to the NF-κB-binding sequences of the *MUC5AC* promoter, and leads to up-regulation of MUC5AC expression in human bronchial epithelial cells [11, 12]. The intracellular Ca^2+^ ([Ca^2+^]_i_) signal is required for activation of NF-κB [13, 14] and subsequently regulates downstream gene expression. But how Ca^2+^ signal is triggered under pathophysiological conditions remains poorly understood.

Transient receptor potential canonical 1 (TRPC1) is a transmembrane protein expressed in a range of vertebrate cells. TRPC1 channel has been shown to contribute to agonist-stimulated Ca^2+^ influx in salivary cells and pancreatic acinar cells [15–17]. Extensive studies have confirmed the contribution of TRPC1 to store-operated Ca^2+^ entry (SOCE). The interaction between TRPC1 and the key components of SOCE, STIM1, and Orai1 determines the activation of TRPC1 [18]. TRPC1 is sub-cellular compartmentalised, at least in part in cholesterol-rich caveolae.

Plasma membrane lipid rafts domains (LRD), which contain high concentrations of cholesterol and sphingolipids, are known to function as centers for the assembly of signaling complexes. Such assembly is known to regulate cellular functions such as transcytosis, protein sorting, cell adhesion and migration as so on. Lipid rafts aggregation facilitate the formation of the STIM1-Orai1-TRPC1 complex and the activation of SOCE [19]. Caveolin-1, a cholesterol-binding protein is involved in the generation of caveolar lipid rafts. The agonist-stimulated Ca^2+^ signals have been shown to originate at caveolin-1 enriched plasma membrane regions [20–23]. In asthma, there are increases in the number of mucus-producing goblet cells in airway epithelium [24]. The bronchial epithelial cell (BEC) is also one of important cell types that produce mucus production [25]. However, the role of lipid rafts/TRPC1 and Ca^2+^ influx in BECs of asthma is unknown.

We hypothesized here that IL-4 increased BECs MUC5AC gene expression through lipid rafts aggregation and TRPC1 channel activation. In the current study, we found that IL-4 caused caveolin-1 containing lipid rafts aggregation and TPRC1 colocalization, and disruption of lipid rafts prevented IL-4 induced NF-κB activation and MUC5AC expression.

## Methods

### Reagents and materials

Methyl-β-cyclodextrin (M-βCD), 2-Aminoethyl diphenylborinate **(**2-APB), ethylene glycol tetraacetic acid (EGTA) and Pronase E were purchased from Sigma-Aldrich (St. Louis, MO, USA). Epithelial cell medium was obtained from Cell Biologics (Chicago, IL, USA). IL-4 was obtained from Pepro Tech (Rocky Hill, NJ, USA). Anti-caveolin-1 antibody was purchased from BD Transduction Laboratories (Lexington, KY, USA), rabbit anti-TRPC1 polyclonal antibody was purchased from Abcam (Cambridge, UK), anti-P65 antibody was purchased from Santa Cruz Biotechnology (Santa Cruz, CA, USA). Cholera toxin subunit B-Alexa Fluor 488 and Trizol Reagent were obtained from Life Technology (Grand Island, NY, USA). Fura-2-AM was purchased from Dojindo laboratories (Kumamoto, Japan). MUC5AC ELISA kit was obtained from Cloud-Clone Corp (Wuhan, China). Anti-MUC5AC antibody was purchased from Absin (Shanghai, China). Antibodies against β-actin, p-STAT6 and p-P65 were purchased from Cell Signaling Technology (Danvers, MA, USA). Specific STAT6 inhibitor AS1517499 and selective IκB kinase (IKK) inhibitor IKK16 was purchased from MedChem Express (Monmouth Junction, NJ, USA).

### Isolation and primary culture of rat BECs

BECs were isolated from large bronchial airway of rat as described in the literature [26]. The experiments were performed in accordance with the Guide for the Care and Use of Laboratory Animals and approved by the Institutional Animal Care and Use Committee (IACUC) of the Tongji Medical College, Huazhong University of Science and Technology. In brief, airway was isolated from rat under sterile conditions, rinsed with ice D-Hanks twice, then digested with 1% Pronase E in DMEM/F-12 at 4°C for 14 h. Then BECs were harvested with ice D-hanks contain 5% newborn bovine serum (NBS) and centrifuged at 1000 rpm for 5 min. The spun down cells were resuspended with epithelial cell medium (Cell Biologics, Chicago, IL, USA) and incubated in a 5% CO_2_ incubator (Thermo Fisher Forma, Waltham, MA, USA) at 37 °C. These cells were used in experiments without subculture. In most experiments, BECs were treated by the indicated factors in serum-free medium when the cells were 60–70% confluence. For MUC5AC immunostaining, the isolated cells digested with protease were cultured on cover slip with serum-containing medium for 24 h in the Petri dish. Some digested cells were presented as small clusters, but not completely separated cells. IL-4 containing serum-free medium was administered when the cells reached about 30% confluence.

### [Ca^2+^]_i_ measurements

BECs [Ca^2+^]_i_ were measured after loading with 10 μM of the acetoxymethyl ester form of Fura-2 for 30 min at room temperature. Then, the fluorescence of Fura-2 was recorded with a stimulation of 20 ng/ml IL-4, in the presence or absence of 10 mM M-βCD or 50 μM 2-APB after excitation at 340 ± 10 and 380 ± 10 nm using a xenon short-arc lamp (Ushio). Bandpass interference filters (Omega Optical, Brattleboro, VT 05301) selected wavelength bands of emitted fluorescence at 510 ± 10 nm. In the process of [Ca^2+^]_i_ measurements, we firstly got stable [Ca^2+^]_i_ signal, 250 s later, we treated cells with 2-APB or IL-4. M-βCD was used before [Ca^2+^]_i_ measurements. In some experiments, we firstly treated cells with 2-APB or M-βCD, then added IL-4 to the cells at 1800s. Emitted Fura-2 fluorescence was collected and measured using a spectrofluorometer (PTI, Deltascan).

To acquire the final [Ca^2+^]_i_ with the ratios from Fura-2 fluorescence, we had detected the minimum and maximum Fura-2 fluorescence ratio (R_min_ and R_max_) with Ca^2+^-free buffer without calcium (8.182 g NaCl, 0.335 g KCl, 0.385 g EGTA, 2 g Glucose, 1.2 g HEPES, 0.2 g MgCl_2_, 1 L H_2_O, PH7.4) and Ca^2+^-high buffer with high concentration of Ca^2+^ (8.182 g NaCl, 0.335 g KCl, 0.555 g CaCl_2_, 2 g Glucose, 1.2 g HEPES, 0.2 g MgCl_2_, 1 L H_2_O, PH7.4). The R_min_ was determined using Ca^2+^-free buffer and stimulated by 3 μM ionomycin (Sigma). The same to R_min,_ the R_max_ was measured with Ca^2+^-high buffer and stimulated by 3 μM ionomycin. The final [Ca^2+^]_i_ was calculated with R_min_ and R_max_ follow this equation: [Ca^2+^]_i_ = Kd*β*(R – R_min_)/(R_max_ – R), Kd is a constant which is 224 for BECs, and the β values is the fluorescence intensity emitted by 380 ± 10 nm within Ca^2+^-free buffer. Ca^2+^ influx duration was calculated from the beginning of Ca^2+^ level rise to the time of Ca^2+^ level return to the base.

### NF-κB activity assay

An ELISA-based assay was performed to measure endogenous NF-κB activities as described previously [27]. Briefly, after BECs were treated with 20 ng/ml IL-4 for 24 h in the presence or absence of M-βCD or 2-APB (M-βCD or 2-APB was administrated half an hour before IL-4 using), the cells were lysed with lysis buffer (20 mM HEPES pH 7.5, 0.35 M NaCl, 20% glycerol, 1% NP-40, 1 mM MgCl_2_·6H_2_O, 0.5 mM EDTA, 0.1 mM EGTA) containing a protease inhibitor cocktail (Calbiochem, La Jolla, CA, USA) on ice for 10 min. The supernatant obtained after centrifugation at 14,000 rpm for 30 min at 4 °C was recovered. The double-stranded probe with single-stranded-linker was generated by 1:1 mix of the following two oligonucleotide with the sequences of 5′-AGTTGAG*GGG*ACTTTCCCAGGCC-(C)34-C-3′, the 3′ end biotinylated and 5′-GCCTGGGAAAGTCCCCTCAACT-3′, respectively. The probe was denatured at 94 °C for 10 min, annealed at room temperature overnight and then linked to streptavidin-coated 96-well plates (Roche, Mannheim, Germany) by incubating 2 pmol of probe per well for 1 h at 37 °C in 50 μl PBS. After wash, 20 μl of cell extract were mixed with 30 μl of binding buffer (4 mM HEPES pH 7.5, 100 mM KCl, 8% glycerol, 5 mM DTT, 0.2% BSA, 40 μg/ml salmon sperm DNA) in the above microwells incubated at room temperature with mild agitation (200 rpm) for 1 h. After wash, mouse anti-NF-κB p65 monoclonal antibody (1:1000 diluted) was incubated for 1 h at room temperature. After wash, peroxidase-conjugated goat anti-mouse IgG were incubated at room temperature for 1 h. After wash, 100 μl tetramethylbenzidine was incubated at room temperature for 10 min before adding 100 μl of stopping solution (2 M H_2_SO_4_). Optical density was then read at 450 nm under a microplate reader (Biotek Instruments, Winooski, VT, USA) using a 655-nm reference wavelength. Backgrounds are determined in lysis buffer and subtracted before data analysis.

### Real-time quantitative RT-PCR (qRT-PCR) analysis for MUC5AC mRNA levels

Total RNA was extracted from BECs using Trizol Reagent. cDNAs were synthesized from total RNA by reverse transcription according to the manufacturer’s instruction. Then cDNAs were used for amplification by qRT-PCR in a 25 μl reaction using SYBR® Premix EX Taq™ II (TaKaRa) with a Mini Opticon Real-time PCR Systerm (BIORAD). MUC5AC mRNA expression was normalized with β-actin. The MUC5AC primers were F: 3-GCTCATCCTAA GCGACGTCT-5, R: 3-GGGGGCATAACTTCTCTTGG-5, and the β-actin primers were F: 3-CGGCATTGTCACCAACTG-5, R: 3-CGCTCGGTCAGGATCTTC-5.

### ELISA analysis for MUC5AC protein levels

Intracellular MUC5AC protein levels were measure using ELISA kits following the manufacturer’s instructions (Cloud-Clone Corp, Wuhan, China).

### Immunofluorescence staining and confocal microscopy

To determine the protein level of MUC5AC, BECs were cultured on cover slips, after pretreated with EGTA (0.5 mM, 2 mM or 5 mM), AS1517499 or LKK16 for 30 min, cells were treated with IL-4 (20 ng/ml). 24 h later, BECs were stained with rabbit anti-MUC5AC antibody (1:100 dilution) at 4 °C overnight, and then incubated with tetramethyl rhodamin isothiocyanate (TRITC)-conjugated goat anti-rabbit antibody at room temperature for 60 min. The nuclei were stained for DAPI for 10 min in dark. The fluorescence-labeled cells were examined using a Zeiss-LSM780 Confocal laser scanning microscope (Oberkochen, German).

To determine the protein level of caveolin-1 and TRPC1, as well as lipid rafts aggregation, BECs were cultured on cover slips, after pretreated with 10 mM M-βCD or 100 μM 2-APB for 30 min, cells were treated with 20 ng/ml IL-4 for 15 min in the presence or absence of M-βCD or 2-APB, BECs were stained with a mouse monoclonal antibody specific to caveolin-1 and rabbit anti-TRPC1 polyclonal antibody. Lipid rafts were visualized by staining with 2 μg/ml cholera toxin subunit B- Alexa Fluor 488 for 20 min.

### Flotation of lipid rafts by sucrose gradient centrifugation

To isolate lipid raft fractions from the cell membrane, BECs were lysed in 1.5 ml buffer containing 10 mM Tris·HCl, 150 mM NaCl, 5 mM EDTA, 1 mMPMSF, 3 mM Na_3_VO_4_, protease inhibitors cocktail and 1% Triton X-100 (pH 7.4). Cell extracts were homogenized with five passages through a 25-gauge needle. Homogenates were adjusted with 60% sucrose density gradient medium to 40% and overlaid with 2 ml 90% sucrose, 4 ml 35% sucrose and 4 ml 5% sucrose Density Gradient medium gently.

Samples were centrifuged at 39000 rpm and 4°C for 18 h using a SW32.1 rotor. Fractions were collected from top to bottom, each sample had 11 fractions. For immunoblot analysis of lipid raft-associated proteins, these fractions were precipitated by mixing with equal volume of 100% trichloroacetic acid and 30 min of incubation on ice, and then samples were centrifuged at 15000 g and 4°C for 15 min, protein sediments were washed with ice acetone twice, air dried, and then resuspended in 1 M Tris·HCl (pH 8.0), which was ready for immunoblot analysis. Western blots were performed to detect protein levels of caveolin-1 and TRPC1 in each fraction.

### Statistical analysis

Results are shown as the mean ± SD for n experiments, n means cells from n rats. Differences between groups were analyzed using unpaired *t* tests or two-way analysis of variance. A *P* value less than 0.05 was considered to be statistically significant.

## Results

### 2-APB prevented IL-4-induced Ca^2+^ influx in BECs

**Fig. 1:**
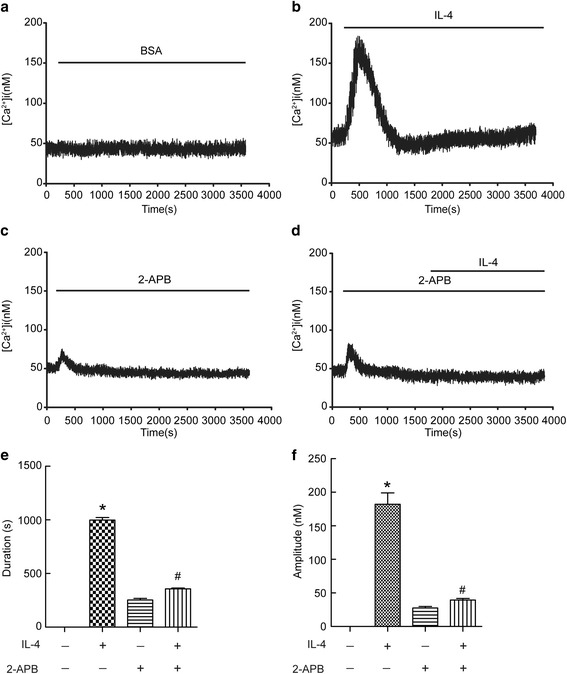
2-APB prevented IL-4-induced Ca^2+^ influx in BECs. Intracellular Ca^2+^ ([Ca^2+^]_i_) levels were recorded using Fura-2/AM. The fluorescence of Fura-2 was detected in cells with a stimulation of IL-4 (20 ng/ml) in the presence or absence of 50 μM 2-APB. Ca^2+^ signals were recorded for the calculation of fluorescence intensity ratio of F340/F380. (**a-d**) Representative traces of calcium responses. (**e** and **f**) Changes in duration and amplitude of [Ca^2+^]_i_. Results are expressed as mean ± SD; *n* = 10. *P < 0.05 versus BSA, ^#^ *P* < 0.05 versus IL-4 treated group

To study the effect of IL-4 on [Ca^2+^]_i_ in BECs, we treated BECs with IL-4 in the presence or absence of 2-APB, an inhibitor of Ca^2+^ release. As shown in Fig. 1a-d, IL-4 significantly increased the level of [Ca^2+^]_i_, but this was abolished by 2-APB. After careful analysis of Ca^2+^ influx duration and amplitude, we found both Ca^2+^ influx duration and amplitude were increased with the treatment of IL-4 in BECs (Fig. 1e, f). These data suggested that IL-4 induced Ca^2+^ influx could be prevented by 2-APB in BECs.

### Extracellular Ca^2+^ signal mediated IL-4-induced MUC5AC protein synthesis in BECs

**Fig. 2:**
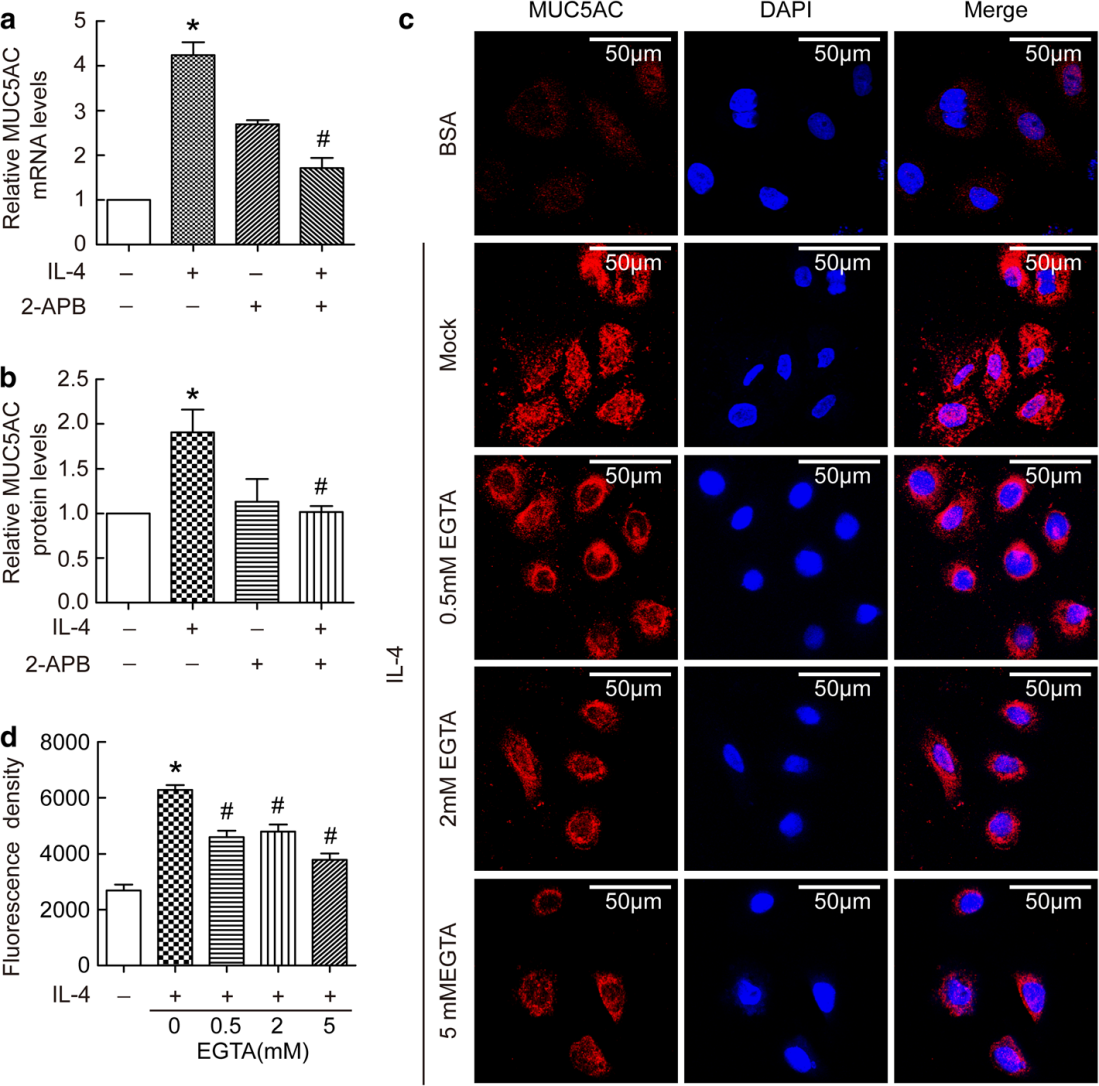
Extracellular Ca^2+^ signal mediated IL-4-induced MUC5AC protein synthesis in BECs. (**a** and **b**) BECs were treated with 20 ng/ml IL-4 for 24 h in the presence or absence of 50 μM 2-APB. Then cell lysates were prepared for the determination of intracellular MUC5AC mRNA and protein levels. Results are expressed as mean ± SD; *n* = 4 experiments. *P < 0.05 versus normal control, ^#^ *P* < 0.05 versus IL-4 treated group. (**c** and **d**) BECs were pretreated with 0 mM, 0.5 mM, 2 mM, or 5 mM EGTA for half an hour, and then treated for 24 h in the presence or absence of 20 ng/ml IL-4. Immunofluorescence staining of MUC5AC protein was performed according to the Methods. Representative staining is shown in the **c**, and immunofluorescence density is shown in the **d**. Results are expressed as mean ± SD; *n* = 21 (cells). *P < 0.05 versus normal control, ^#^ *P* < 0.05 versus IL-4 treated group

To investigate the role of Ca^2+^ influx in MUC5AC synthesis, we detected MUC5AC mRNA and protein levels in BECs treated by IL-4. As shown in Fig. 2a and b, IL-4 increased the levels of MUC5AC mRNA and protein expression, and these were prevented by 2-APB (Fig. 2a, b). Because 2-ABP is nonspecific and blocks both TRP channels and intracellular inositol triphosphate receptor (IP3R), to distinguish between extracellular and intracellular Ca^2+^ source, we blocked extracellular Ca^2+^ using EGTA. As shown in Fig. 2c and d, IL-4 induced increases in MUC5AC expression, but this was attenuated by EGTA at a dose-dependent manner. These data suggested extracellular Ca^2+^ influx played a role in IL-4 induced MUC5AC expression.

### NF-κB rather than STAT6 activation mediated IL-4-induced MUC5AC protein synthesis

**Fig. 3:**
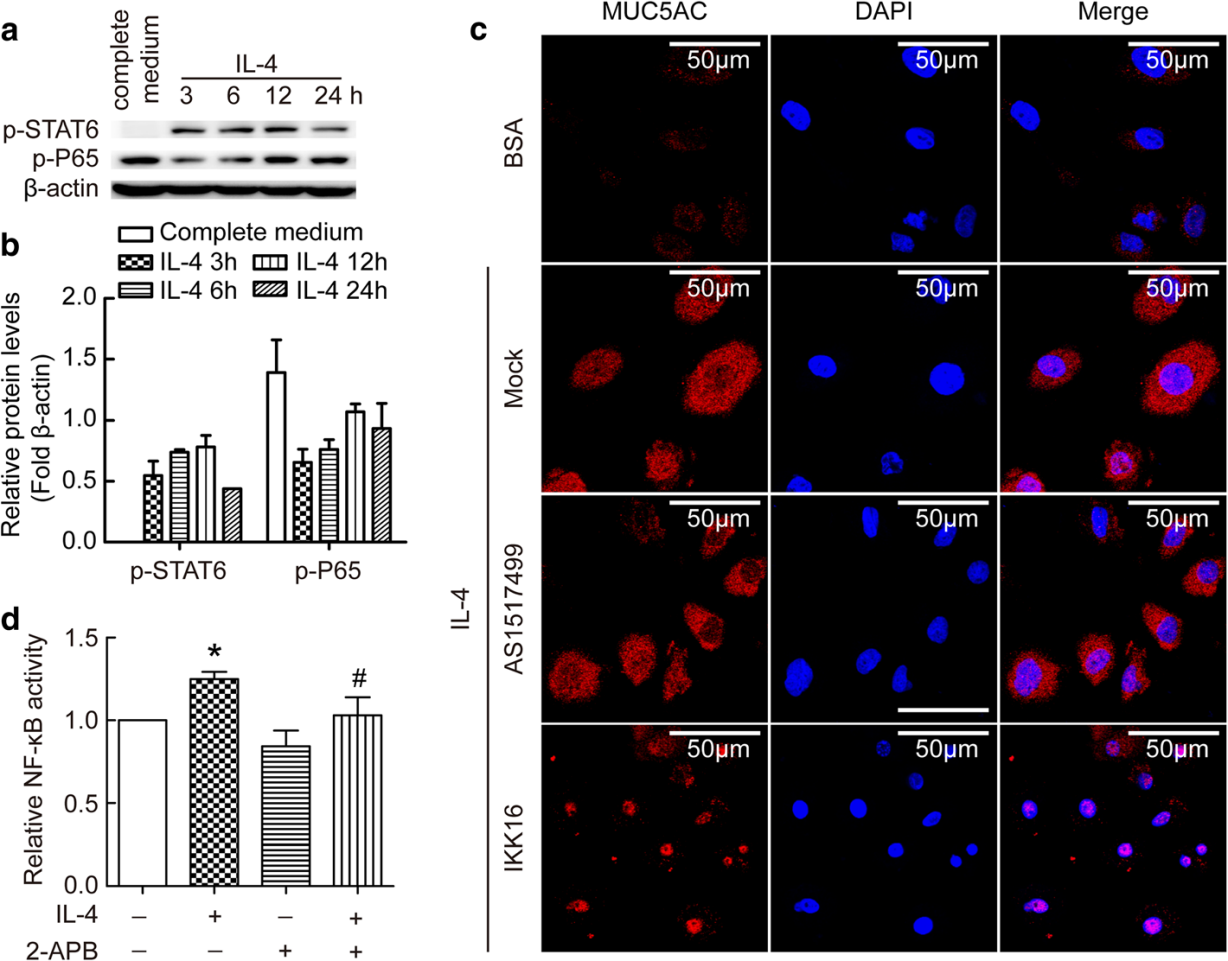
NF-κB rather than STAT6 activation mediated IL-4-induced MUC5AC protein synthesis in BECs. BECs were treated with IL-4 (20 ng/ml) for 3, 6, 12, or 24 h. The complete cell-cultured medium was used as control. Intracellular p-STAT6 and p-P56 proteins were measured by Western blot. (**a**) Representative image of immunoblots of p-STAT6 and p-P56. (**b**) Bar graph depicting changes in relative density of p-STAT6 and p-P56 according to **a**. (**c**) BECs were pretreated with specific STAT6 inhibitor (AS1517499) or a selective IκB kinase inhibitor (IKK 16) for half an hour, then treated for 24 h in the presence or absence of 20 ng/ml IL-4. Immunofluorescence staining of MUC5AC protein was performed according to the Methods. Representative staining is shown in the **c**. (**d**) BECs were treated with 20 ng/ml IL-4 for 24 h in the presence or absence of 50 μM 2-APB. Then cell lysates were used to detect endogenous NF-κB transcriptional activity. Results are expressed as mean ± SD; *n* = 4 experiments. *P < 0.05 versus normal control, ^#^ *P* < 0.05 versus IL-4 treated group

To study downstream signals of IL-4 in BECs, we investigate STAT6 and NF-κB pathways. As shown in Fig. 3a and b, IL-4 induced time-dependent phosphorylation of STAT6 and P65 which suggested both signals were activated in IL-4 treated BECs. To further distinguish the role of STAT6 and NF-kB pathway in IL-4-induced MUC5AC expression, we used specific STAT6 inhibitor AS1517499 and a selective IκB kinase (IKK) inhibitor IKK 16 to treat cells. As shown in Fig. 3c, IKK 16 induced significant down-regulation of MUC5AC protein, but AS1517499 had no effect on IL-4 induced up-regulation of MUC5AC protein. These data suggested that NF-κB rather than STAT6 signal pathway mediated MUC5AC up-regulation induced by IL-4. To further dissect the role of NF-κB as well as the relationship with Ca^2+^ influx, we detected NF-κB activity. As shown in Fig. 3d, IL-4 enhanced NF-κB activity, and this was blocked by 2-APB. These data suggested extracellular Ca^2+^ influx played a role in IL-4 induced NF-κB activation and further MUC5AC expression.

### Caveolin-1 containing lipid rafts aggregation was involved in TRPC1 activation by IL-4 in BECs

**Fig. 4:**
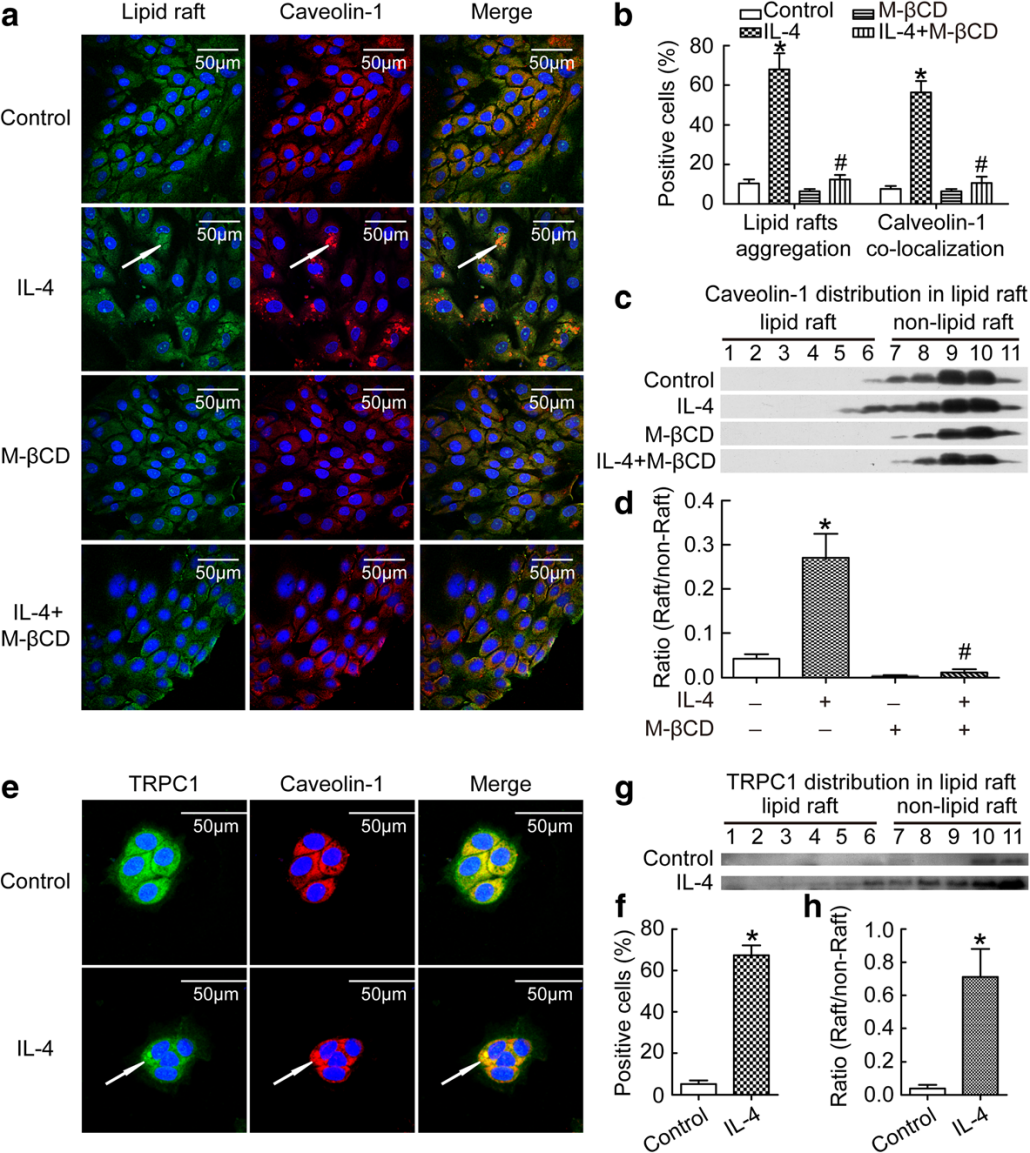
IL-4 induced caveolin-1-containing lipid rafts aggregation and TRPC1 activation. BECs were treated with 20 ng/ml IL-4 for 24 h, then intracellular co-localization of caveolin-1 and lipid rafts were detected with immunofluorescence (**a**); the percents of positive lipid rafts aggregation cells and caveolin-1 co-localization cells were calculated (**b**); caveolin-1 distribution in lipid raft were measured by sucrose gradient centrifugation (**c**); the ratio of caveolin-1 distribution in lipid raft to non-lipid raft was evaluated (**d**); intracellular co-localization of caveolin-1 and TRPC1 were detected with immunofluorescence (**e**); the percents of positive TRPC1 and caveolin-1 co-localization cells were calculated (**f**); TRPC1 distribution in lipid raft was measured by sucrose gradient centrifugation (**g**); the ratio of TRPC1 distribution in lipid raft to non-lipid raft was evaluated (**h**). White arrow (in **a** and **e)** indicated lipid rafts aggregation, calveolin-1 aggregation, or TRPC1 aggregation as well as their co-localization respectively. Results are expressed as mean ± SD; *n* = 4 experiments. *P < 0.05 versus normal control, ^#^ *P* < 0.05 versus IL-4 treated group

To explore the potential mechanism underlying extracellular Ca^2+^ influx induced by IL-4, we next focused on the lipid rafts clustering in BECs. We detected the colocalization and clustering of caveolin-1 and ganglioside GM1 enriched lipid rafts. Cells were first incubated with an anti-calveolin-1 antibody, followed by staining with cholera toxin subunit B-Alexa Fluor 488, which is a specific clustering agent for GM1 enriched lipid rafts. As shown in Fig. 4a and b, IL-4 induced clustering and colocalization of GM1 enriched lipid rafts and caveolin-1. Moreover, when the cells were treated with the inducer of lipid rafts disruption (M-βCD), IL-4 failed to induce clustering of lipid rafts. By using sucrose gradient ultracentrifugation and immunoblotting, lysates were separated into lipid raft and non-lipid raft fractions. As shown in Fig. 4c, caveolin-1 was mainly found in non-lipid raft fractions in the control. However, the treatment of IL-4 led to significantly increased caveolin-1 in lipid raft fractions, and this was prevented by M-βCD (Fig. 4c, d). These data suggested that IL-4 induced caveolin-1 containing lipid rafts aggregation.

TRPC activation is necessary in extracellular Ca^2+^ influx when IP3 binds with IP3R, so to further uncover the relationship between extracellular Ca^2+^ influx and caveolin-1-containing lipid rafts aggregation, we studied the colocalization of TRPC1 and caveolin-1. As shown in Fig. 4e and f, IL-4 induced clustering and colocalization of caveolin-1 and TRPC1. After sucrose gradient ultracentrifugation, the immunoblotting images revealed that TRPC1 was found in non-lipid raft fractions in normal control, but IL-4 caused significant increase of TRPC1 in lipid raft fractions (Fig. 4g, h). These data suggested that IL-4 induced colocalization of TRPC1 in caveolin-1-containing lipid rafts aggregation.

### Blocking lipid rafts aggregation prevented extracellular Ca^2+^ influx induced by IL-4

**Fig. 5:**
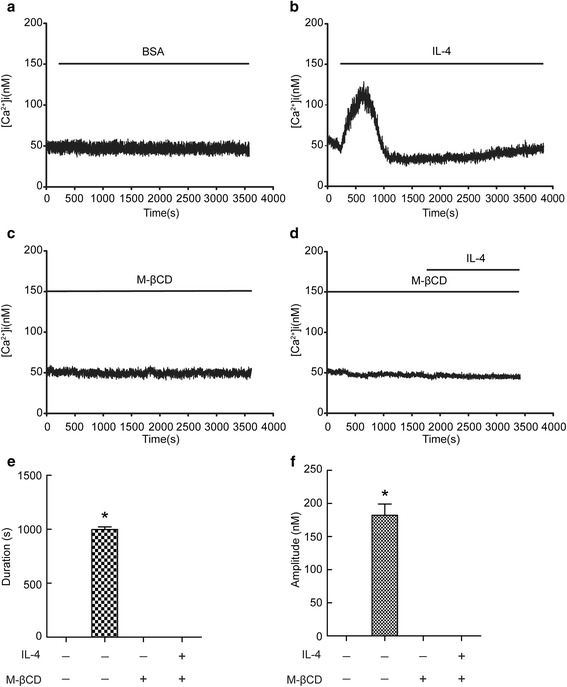
Blocking lipid rafts aggregation prevented Ca^2+^ influx induced by IL-4. Intracellular Ca^2+^ levels were recorded using Fura-2/AM. BECs were pretreated with or without 10 mM M-βCD. Then, the fluorescence of Fura-2 was detected in cells with a stimulation of 20 ng/ml IL-4. Ca^2+^ signals were recorded for calculating the fluorescence intensity ratio of F340/F380. (**a-d**) Representative trace of calcium responses. (**e** and **f**) Changes in Ca^2+^ influx duration and amplitude. Results are expressed as mean ± SD; *n* = 12. *P < 0.05 versus BSA, ^#^ *P* < 0.05 versus IL-4 treated group

To further confirm the colocalization of TRPC1 in caveolin-1-containing lipid rafts aggregation, we detected [Ca^2+^]_i_ levels. BECs were exposed to IL-4 in the presence or absence of M-βCD. As shown in Fig. 5, IL-4 triggered increases in [Ca^2+^]_i_ including amplitude and duration, but after disruption of lipid raft clustering with M-βCD, IL-4 did not induce any change in [Ca^2+^]_i_ signal. These data suggested that IL-4 induced intracellular calcium response is mediated by lipid rafts aggregation.

### Caveolin-1-containing lipid rafts aggregation induced by IL-4 contributed to MUC5AC synthesis

**Fig. 6:**
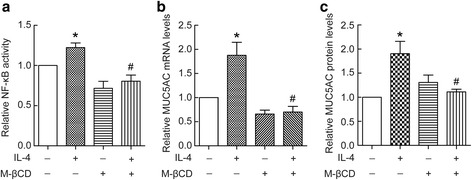
Caveolin-1-containing lipid rafts aggregation by IL-4 contributed to MUC5AC synthesis. BECs were treated with 20 ng/ml IL-4 for 24 h in the presence or absence of 10 mM M-βCD. Then, cell lysates were prepared for determination of endogenous NF-κB transcriptional activity (**a**), and intracellular MUC5AC mRNA and protein levels (**b** and **c**). Results are expressed as mean ± SD; *n* = 4 experiments. *P < 0.05 versus normal control, ^#^ *P* < 0.05 versus IL-4 treated group

To study the role of IL-4-induced caveolin-1-containing lipid rafts aggregation in MUC5AC synthesis in BECs, we used M-βCD to disrupt lipid rafts aggregation, and then detected changes in NF-κB activity and MUC5AC levels. As shown in Fig. 6a, IL-4 induced increases in NF-κB activity, and this was blocked by M-βCD. IL-4 significantly increased the intracellular MUC5AC mRNA and protein levels (Fig. 6b, c), disruption of lipid raft by M-βCD attenuated IL-4-induced increases of MUC5AC. These data suggest that IL-4 induced caveolin-1-containing lipid rafts aggregation which contributed to MUC5AC synthesis.

## Discussion

**Fig. 7:**
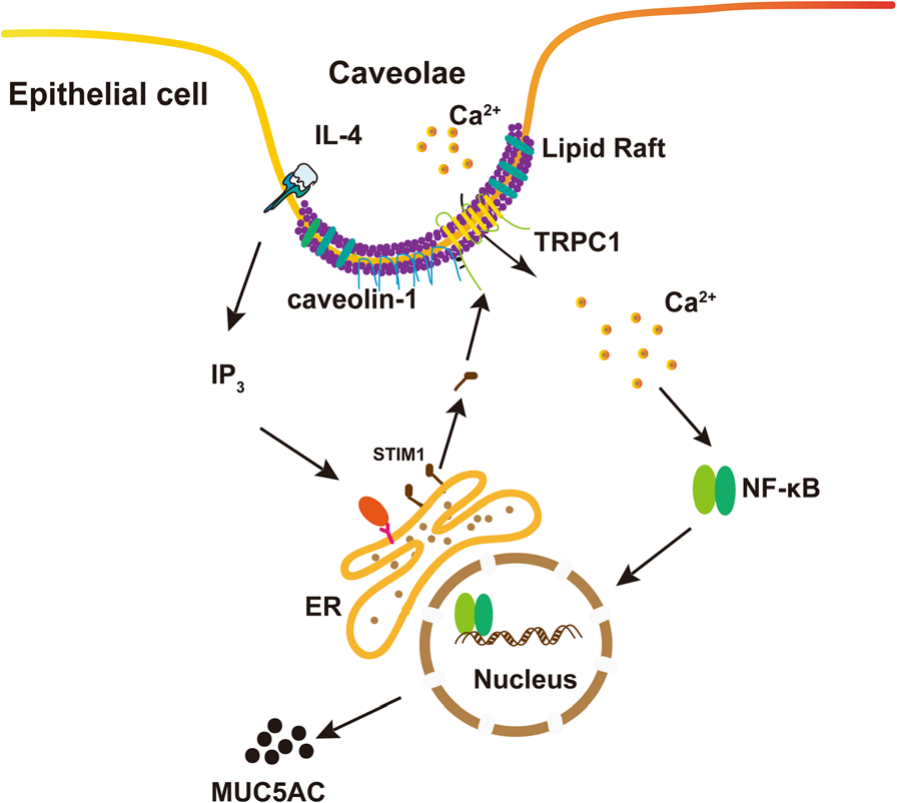
IL-4 induced MUC5AC overproduction via cell membrane lipid rafts aggregation and activation of TRPC1

In this study, we provided substantial evidence linking asthmatic cytokine IL-4 and airway epithelial cells MUC5AC overproduction. Our additional data revealed that MUC5AC proteins were over-expressed in asthmatic rat airway epithelial cells in vivo (Additional file 1). We found that IL-4 induced caveolin-1-containing lipid rafts aggregation, and co-localization with TRPC1 in the lipid rafts domain. Disruption of lipid rafts or blocking of TRPC1 attenuated IL-4 induced calcium signals and NF-κB activation, consequently prevented MUC5AC over-expression. Thus, these observations indicated that IL-4 induced MUC5AC overproduction via cell membrane lipid rafts aggregation and activation of TRPC1 (Fig. 7).

In the current study, firstly we found IL-4 induced Ca^2+^ influx and MUC5AC overproduction in BECs which prevented by 2-APB. Because 2-ABP is nonspecific and blocks both TRP channels and IP3R, to distinguish between extracellular and intracellular Ca^2+^ source, we depleted extracellular Ca^2+^ using EGTA and found extracellular Ca^2+^ influx played a role in IL-4 induced MUC5AC expression. We next found NF-κB rather than STAT6 activation mediated IL-4-induced MUC5AC protein synthesis.

For the first time, we found that IL-4 induced caveolin-1-containing lipid rafts aggregation in BECs. Membrane lipid rafts are highly ordered membrane domains that are enriched in cholesterol, sphingolipids and gangliosides, and function as platforms for the recruitment of signaling proteins to facilitate protein-protein interaction and signal transduction. An increasing number of proteins involved in signal transduction have been found to locate in these ordered membrane domains. It is well known that lipid rafts organize receptors, ion channels and their downstream acting molecules to regulate intracellular signaling pathways [28–30].

Recent studies have demonstrated that lipids rafts also contribute to the organization and function of Ca^2+^ signaling microdomains. TRPC1 channelosome responsible for SOCE is located in caveolar lipid raft domains [18, 31–33]. Destabilization of the caveolar lipid raft domains by M-βCD treatment or deletion of caveolin-1 prevents SOCE activation [20, 34]. Caveolin-1 gene knockout disrupts TRPC1-STIM1-Orai1 complex [35]. In the current study, we demonstrated that IL-4 induced TRPC1 co-localization with caveolin-1-containing lipid rafts aggregation, which mediated Ca^2+^ influx in BECs.

Calcium signal was observed to be related with NF-κB activation. Sequence analyses of MUC5AC promoter revealed the presence of NF-κB response elements within it [36]. NF-κB activity is crucial for mucus production [37] . Zhu et al. have reported that Ca^2+^ oscillation frequency regulated NF-κB transcriptional activity via increased cumulated Ca^2+^ spike duration [38, 39]. Our data showed here that IL-4 induced single calcium peak, blocking TRPC1 with 2-APB prevented calcium signal and suppressed NF-κB activity. These data suggest that IL-4 induced calcium signaling through caveolin-1-containing-lipid-rafts/TRPC1 pathway that resulted in NF-κB activation and MUC5AC over-production.

## Conclusion

In the current study, we found IL-4 caused aggregation of caveolin-1-containing lipid rafts which activated TPRC1, subsequently activated calcium signal and NF-κB, and finally increased MUC5AC synthesis in BECs. This study elucidates a new mechanism underlying asthmatic cytokines induce mucus overproduction, which may be beneficial in developing novel strategies for the treatment of asthma.

## Additional file

Additional file 1: Online supplementary data. (PDF 316 kb)

## Abbreviations

[Ca^2+^]_i_: intracellular Ca^2+^
2-APB: 2-Aminoethyl diphenylborinate
BECs: Bronchial epithelial cells
EGTA: Ethylene glycol tetraacetic acid
IL-4: Interleukin-4
IP3R: Inositol triphosphate receptor
M-βCD: Methyl-β-cyclodextrin
SOCE: Store-operated Ca^2+^ entry
Th2: T helper type 2 cells
TRPC1: Transient receptor potential canonical 1
