# Supplementary material for: IL-4-induced caveolin-1-containing lipid rafts aggregation contributes to MUC5AC synthesis in bronchial epithelial cells
Source: Respir Res. 2017 Sep 20;18:174. doi: 10.1186/s12931-017-0657-z (PMC5607571; doi:10.1186/s12931-017-0657-z)
Supplement: Additional file 1: — Online supplementary data. (PDF 316 kb) [file 12931_2017_657_MOESM1_ESM.pdf]

Online supplementary data

Title： IL-4-induced caveolin-1-containing lipid rafts aggregation contributes to MUC5AC synthesis in bronchial epithelial cells

Authors： Yu Xia, *et al*.

Supplementary methods

Asthma animal model

The rat model was generated by ovalbumin (OVA) sensitization and challenge as previously described with some modifications [1]. This model demonstrates many features of airway allergy and allergic asthma that are similar to human disease. 4-week-old male Sprague-Dawley rats (150 ± 20 g) were housed in the Experimental Animal Center of Tongji Medical College with *ad libitum* access to food and water. The study protocol was approved by the Institutional Animal Care and Use Committee of Tongji Medical College, Huazhong University of Science and Technology. Rats were sensitized with intraperitoneal injection of 10 mg OVA (Grade V, Sigma, St. Louis, MO, USA) plus 2 mg aluminum hydroxide in 1 ml saline on days 1 and 8. Between days 15 to 75, aerosol challenges containing either 1% OVA or saline were given to rats (for 20 minutes, three times a week). Rats were euthanized at days 75.

Immunofluorescence staining of lung tissue

The lungs were removed after thoracotomy. Lung tissues were fixed with 4% paraformaldehyde for 2 days at room temperature, and embedded in paraffin. The slides were

incubated with rabbit anti-MUC5AC antibody (1:100 dilution) at 4°C overnight and then incubated with tetramethyl rhodamin isothiocyanate (TRITC)-conjugated goat anti-rabbit antibody for 50 min. The nuclei were stained for DAPI for 10 minutes in dark. The slides were mounted with anti-fade reagent and examined using a Zeiss-LSM780 Confocal laser scanning microscope (Oberkochen, German).

Supplementary results

IL-4 induced increases in MUC5AC protein in rat bronchial epithelial cells (BECs)

BECs were isolated from large bronchial airway of rat. To confirm these primarily BECs can produce MUC5AC protein in the presence of IL-4, we treated the cells with IL-4, then performed immunofluorescence staining with anti-MUC5AC antibody. As shown in Fig. S1a, the control cells had very little MUC5AC protein expression, but IL-4 induced significant increases in MUC5AC protein. To further confirm our results *in vivo*, we stained lung tissues of rat asthma model with anti-MUC5AC antibody, expression of MUC5AC proteins in the large airway (Fig. S1b) and moderate airway (Fig. S1c) were obviously stronger than that in control rats. So, our *in vitro* and *in vivo* data showed that IL-4 induced increases of MUC5AC protein in primary rat BECs.

Reference

1. Kohan M, Breuer R, Berkman N. Osteopontin induces airway remodeling and lung fibroblast activation in a murine model of asthma. *Am J Respir Cell Mol Biol* 2009; 41:290-6.

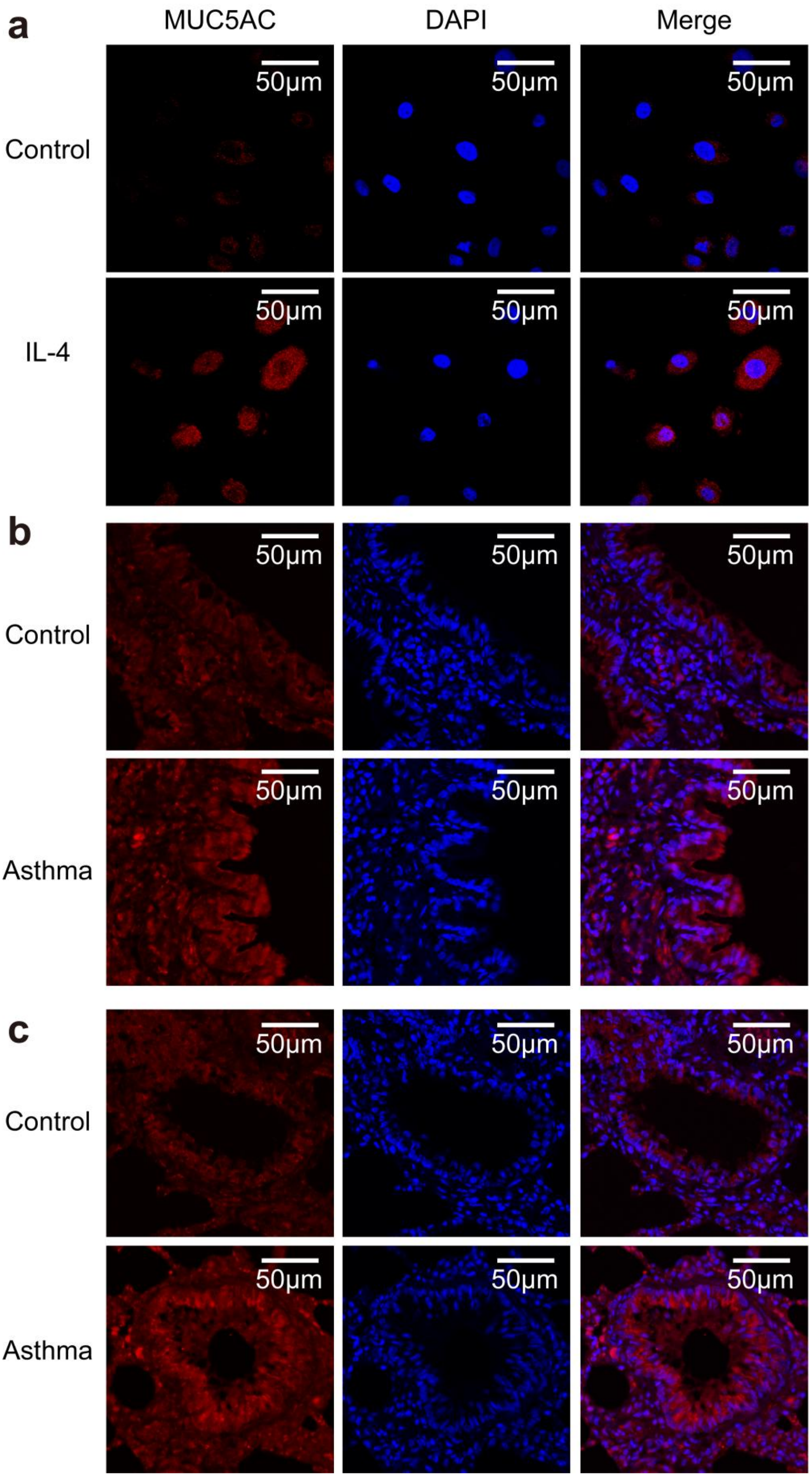

Fig. S1 IL-4 induced increases in MUC5AC protein in rat bronchial epithelial cells. BECs

were treated with 20 ng/ml IL-4 for 24 hours, then stained with anti-MUC5AC antibody as the methods. (a) Representative images of BECs from rat large bronchial airway. (b and c) Asthma animal model and immunofluorescence staining of lung tissue with anti-MUC5AC antibody were performed as the supplementary methods. (b) Representative images of large bronchial airway. (c) Representative images of middle bronchial airway.
